# DeepProSite: structure-aware protein binding site prediction using ESMFold and pretrained language model

**DOI:** 10.1093/bioinformatics/btad718

**Published:** 2023-11-28

**Authors:** Yitian Fang, Yi Jiang, Leyi Wei, Qin Ma, Zhixiang Ren, Qianmu Yuan, Dong-Qing Wei

**Affiliations:** State Key Laboratory of Microbial Metabolism, Shanghai-Islamabad-Belgrade Joint Innovation Center on Antibacterial Resistances, Joint International Research Laboratory of Metabolic & Developmental Sciences and School of Life Sciences and Biotechnology, Shanghai Jiao Tong University, Shanghai 200040, China; Peng Cheng Laboratory, Shenzhen 518055, China; Department of Biomedical Informatics, College of Medicine, The Ohio State University, Columbus, OH 43210, USA; School of Software, Shandong University, Jinan, Shandong 250100, China; Department of Biomedical Informatics, College of Medicine, The Ohio State University, Columbus, OH 43210, USA; Peng Cheng Laboratory, Shenzhen 518055, China; School of Computer Science and Engineering, Sun Yat-sen University, Guangzhou 510000, China; State Key Laboratory of Microbial Metabolism, Shanghai-Islamabad-Belgrade Joint Innovation Center on Antibacterial Resistances, Joint International Research Laboratory of Metabolic & Developmental Sciences and School of Life Sciences and Biotechnology, Shanghai Jiao Tong University, Shanghai 200040, China; Peng Cheng Laboratory, Shenzhen 518055, China

## Abstract

**Motivation:**

Identifying the functional sites of a protein, such as the binding sites of proteins, peptides, or other biological components, is crucial for understanding related biological processes and drug design. However, existing sequence-based methods have limited predictive accuracy, as they only consider sequence-adjacent contextual features and lack structural information.

**Results:**

In this study, DeepProSite is presented as a new framework for identifying protein binding site that utilizes protein structure and sequence information. DeepProSite first generates protein structures from ESMFold and sequence representations from pretrained language models. It then uses Graph Transformer and formulates binding site predictions as graph node classifications. In predicting protein–protein/peptide binding sites, DeepProSite outperforms state-of-the-art sequence- and structure-based methods on most metrics. Moreover, DeepProSite maintains its performance when predicting unbound structures, in contrast to competing structure-based prediction methods. DeepProSite is also extended to the prediction of binding sites for nucleic acids and other ligands, verifying its generalization capability. Finally, an online server for predicting multiple types of residue is established as the implementation of the proposed DeepProSite.

**Availability and implementation:**

The datasets and source codes can be accessed at https://github.com/WeiLab-Biology/DeepProSite. The proposed DeepProSite can be accessed at https://inner.wei-group.net/DeepProSite/.

## 1 Introduction

Interactions between proteins and various biological molecules form the basis of protein function in most biological processes, such as gene expression regulation, signal transduction, and metabolic pathway regulation (Pawson and Nash 2003, Kortemme *et al.* 2004, Hermann *et al.* 2007, Rubinstein and Niv 2009, Babu *et al.* 2011, Zhang *et al.* 2012). These interactions regulate normal cellular functions and play a key role in the pathogenesis of various diseases. For example, in diseases such as tumour growth, autoimmune disorders, and pathogen invasion, the dysregulation of protein–protein interactions can lead to disorders of pathophysiological processes (Clare and Clary 2004, Tovar *et al.* 2006, Penna *et al.* 2007, London *et al.* 2012, Lee *et al.* 2015). Moreover, therapeutic peptides have become a focus of drug development because they can bind to proteins and have therapeutic effects (Fosgerau and Hoffmann 2015, Bruzzoni-Giovanelli *et al.* 2018, Lau and Dunn 2018, Davenport *et al.* 2020). Therefore, understanding the location and characteristics of protein binding sites is crucial for understanding protein function and drug design (Wells and McClendon 2007, De Las Rivas and Fontanillo 2012, Kuzmanov and Emili 2013, Valkov *et al.* 2016, Guilliam *et al.* 2017, Batra *et al.* 2018). Traditional binding site detection methods, such as X-ray crystallography, two-hybrid screening, surface plasmon resonance techniques, and affinity purification-mass spectrometry, are expensive and time-consuming (Orengo *et al.* 1997, Shoemaker and Panchenko 2007, Terentiev *et al.* 2009, Brettner and Masel 2012, Wodak *et al.* 2013). In addition, several technical challenges, including the small size of peptides (Vlieghe *et al.* 2010), weak binding affinity (Dyson and Wright 2005), conformational flexibility (Bertolazzi *et al.* 2014), high transience, and dynamics of protein–protein interactions, increase the difficulty in accurately identifying the binding residues. Therefore, it is important to develop new, fast, and accurate computational methods.

In regard to predicting protein binding sites, computational methods can generally be divided into two categories: those that rely on the protein sequence and those that rely on the protein structure. Sequence-based methods are based on the primary amino acid sequences of proteins for binding site prediction, and some notable examples are DLPred (Zhang *et al.* 2019), ProNA2020 (Qiu *et al.* 2020), DELPHI (Li *et al.* 2021), SPRINT-Seq (Taherzadeh *et al.* 2016), PepBind (Zhao *et al.* 2018), Visual (Wardah *et al.* 2020), PepNN-Seq (Abdin *et al.* 2020), pepBCL (Wang *et al.* 2022), and DNAPred (Zhu *et al.* 2019). Although sequence-based methods allow for the prediction of any protein based solely on its sequence, their accuracy is limited because potential patterns of binding sites are conserved in the spatial structure but are not evident from their sequences (Chen *et al.* 2017, Rigden and Rigden 2009). As a result, features extracted from protein sequences may be insufficient to correctly represent residues. In contrast, methods based on protein structures tend to be more precise when predicting binding sites. The main current structure-based methods are SPPIDER (Porollo and Meller 2007), MaSIF-site (Gainza *et al.* 2020), GraphPPIS (Yuan *et al.* 2021), PepSite (Petsalaki *et al.* 2009), Peptimap (Lavi *et al.* 2013), SPRINT-Str (Taherzadeh *et al.* 2018), PepNN-Struct (Abdin *et al.* 2020), and GraphBind (Xia *et al.* 2021).

Despite the recent progress made using computational methods, their application in large-scale high-throughput prediction is constrained by several issues. First, structure-based methods require known tertiary structures as a prerequisite. However, because the structures of most proteins are still unknown, this approach is not applicable to proteins without a known structure. Moreover, experimental determination of protein structures is a time-consuming and challenging task. Second, sequence-based methods often use evolutionary information, which is typically generated by comparing the query protein to a large-scale protein database in terms of sequence. However, the performance of this approach is poor when predicting proteins with insufficient sequence similarity. Extracting evolutionary features also takes a long time. Third, existing methods rely heavily on hand-designed features to construct models, which may fail to consider key biological features.

In recent years, the field of protein structure prediction has witnessed remarkable advancements due to the application of deep learning techniques. These include AlphaFold2 (Jumper *et al.* 2021) and RoseTTAFold (Baek *et al.* 2021). Among these advances, ESMFold (Lin *et al.* 2023) has emerged as a noteworthy development that deploys a large-scale pretrained protein language model, instead of the conventional multiple sequence alignment method, to generate structure predictions. By utilizing a single sequence as input, ESMFold greatly speeds up the prediction process while maintaining high accuracy in predicting atomic resolution structures. Moreover, ESMFold outperforms other methods when dealing with proteins that have few homologous sequences. This remarkable achievement undoubtedly offers immense potential to contribute to downstream studies on protein structure and function, including binding site prediction.

Simultaneously, graph neural networks and their variants (Chen *et al.* 2021, Yuan *et al.* 2021) have been widely used in various graph-related tasks. However, achieving effective learning for protein structures remains a challenging task. On the other hand, Transformer (Vaswani *et al.* 2017) has rapidly become a mainstream architecture for natural language processing, speech recognition, and protein sequence processing (Devlin *et al.* 2018, Chen *et al.* 2020, Zheng *et al.* 2020). In the past few years, variants of Transformer have excelled in graph representation learning (Ingraham *et al.* 2019, Chen *et al.* 2021, Ying *et al.* 2021). Compared to the Transformer, the Graph Transformer introduces graph topology, has powerful representation capabilities in graph data modelling, captures complex relationships between nodes, can handle different types of nodes and edges, and is applied inside protein representation learning (Ingraham *et al.* 2019). Therefore, the Graph Transformer technology is capable of constructing accurate protein structure models from sequences, effectively learning structural information, and has the potential to improve protein binding site prediction.

In this study, DeepProSite is presented as a topology-aware Graph Transformer model that generates effective structural information and sequence information representations from protein sequences, utilizing ESMFold and pretrained language models, respectively, for predicting protein binding sites. We showcase the capabilities of DeepProSite in predicting protein–protein/peptide binding sites. DeepProSite outperforms state-of-the-art sequence- and structure-based methods. Additionally, this is the first study to utilize ESMFold-predicted structures for protein–protein/peptide binding site prediction. This method is also suitable for predicting the binding sites of nucleic acids and other ligands (such as ATP, HEME, and metal ions) and does not rely on explicit parameterization of any physicochemical properties. Finally, we developed a user-friendly and comprehensive web server for site prediction that can be conveniently accessed and used by biologists.

## 2 Materials and methods

### 2.1 Datasets

In this study, we use the same benchmark datasets as in previous studies to train and evaluate our method. Table 1 provides detailed information about the datasets. Specifically, 1279 peptide-binding proteins (PBPs) were originally derived from the study of SPRINT-Seq (Taherzadeh *et al.* 2016). These datasets were collected from the BioLiP database (Yang *et al.* 2013a) and proteins with more than 30% sequence identity were excluded. Peptide-binding residues in a protein were defined as residues containing at least one atom, with distance less than 3.5 Å from any atom in the peptide. To ensure fair comparison with previous studies, we employ identical data partitioning strategies for both model training and testing. Ten percent of the randomly selected compounds in the study of SPRINT-Str (Taherzadeh *et al.* 2018) are used as the independent test dataset (Pep_Test_125), and the rest are used as the training dataset (Pep_Train_1154). PepBind (Zhao *et al.* 2018) randomly divided these 1279 PBPs into two equally sized subsets for training and testing (Pep_Train_640 and Pep_Test_639).

**Table 1.** Statistics information of the benchmark datasets used in this study.

Type	Dataset	*N*_protein_ ^a^	*N*_pos_ ^b^	*N*_neg_ ^c^	PNratio^d^
Peptide	Pep_Train_1154	1154	15 030	261 792	0.057
	Pep_Test_125	125	1719	29 151	0.059
	Pep_Train_640	640	8259	149 103	0.055
	Pep_Test_639	639	8490	141 840	0.060
Protein	Pro_Train_335	335	10 374	55 992	0.185
	Pro_Test_60	60	2075	11 069	0.187
	Pro_Test_315	315	9355	55 976	0.167

a Number of proteins.

b Number of binding residues.

c Number of nonbinding residues.

d PNratio = *N*_pos_/*N*_neg_.

Three datasets from previous studies [Pro_Dset_186 (Murakami and Mizuguchi 2010), Pro_Dset_164 (Dhole *et al.* 2014), and Pro_Dset_72 (Murakami and Mizuguchi 2010)] were used to predict protein–protein binding sites. These datasets were built from annotated complexes in the Protein Data Bank (PDB) (Berman *et al.* 2000) and the protein–protein docking benchmark set version 3.0 (Hwang *et al.* 2008). In these datasets, a protein-interacting residue was defined as a surface residue (RSA > 5%) that lost more than 1 Å^2^ of absolute solvent accessibility after protein–protein complex formation. A previous study (Yuan *et al.* 2021) integrated three datasets into a fused dataset. Redundant proteins with a sequence similarity of over 25% and sequence coverage greater than 90% were removed, resulting in a final dataset of 395 proteins. Among these proteins, a subset of 335 proteins (designated Pro_Train_335) was randomly selected for training, while the remaining 60 proteins were reserved for independent testing (designated Pro_Test_60). Another independent test dataset (Pro_Test_315) was constructed from protein complexes published in recent years (January 2014–May 2021). Furthermore, the induced fit or conformational selection often results in conformational changes during protein binding (Hammes *et al.* 2009). To assess the robustness of the method and evaluate how conformational changes affect its performance, a previous study (Yuan *et al.* 2021) collected 31 unbound structures corresponding to 31 proteins sourced from Pro_Test_60 to generate an additional unbound test dataset.

### 2.2 Protein graph construction

The protein–protein/peptide binding site prediction in this work is formulated as a node classification task in a protein graph. For a protein with *n* residues, we extracted the sequence and structural features, as well as the 3D coordinates of each node, from which a node feature matrix $H\in R^{n\times d}$ and a coordinate matrix $X\in R^{n\times 3}$ were derived to construct an attributed protein graph.

#### 2.2.1 Predicted protein structures

To capture the geometric information of each residue, we applied esmfold_v1 (Lin *et al.* 2023) (denoted as ESMFold) to predict the structure for a given sequence. ESMFold adopts a large language model and an end-to-end neural network to accurately make atomic resolution structure predictions with no need for multisequence alignment, resulting in up to 60× faster than the state of the art while maintaining similar accuracy. We downloaded the pretrained ESMFold model to predict the structures of our whole binding site datasets mentioned above.

#### 2.2.2 Structural properties

For each residue in the ESMFold-predicted structures, we extracted three types of structural features utilizing DSSP (Kabsch and Sander 1983). The first is relative solvent accessibility (RSA), which provides useful information for the prediction of binding sites since solvent-exposed residues have more potential to interact with other molecules. RSA is normalized from the solvent accessible surface area (ASA) value by the maximal ASA of a specific amino acid type. The second is one-hot secondary structure profile representing eight categories of secondary structure states. The third is sine and cosine values of the protein backbone torsion angles ϕ and ψ reflect the geometry. These three sets of features are combined to form a 14-dimensional feature group, which we refer to as DSSP.

#### 2.2.3 Language model embeddings

We applied the widely used protein language model named ProtT5-XL-U50 (Elnaggar *et al.* 2021) (denoted as ProtT5) to extract sequence information, which is a self-supervised autoencoder based on the transformer model. Specifically, ProtT5 was first pretrained on BFD (Steinegger *et al.* 2019), a dataset containing a large number of protein sequences constructed using block search and combination algorithms, to predict masked amino acids according to the sequence context. Then, ProtT5 was fine-tuned on UniRef50, a dataset containing more than 500 million nonredundant protein sequences covering a variety of biological species and functions (Suzek *et al.* 2015). We extracted a 1024-dimensional sequence embedding for each residue using ProtT5 and normalized them to scores between 0 and 1 using Formula (1):


(1) xnorm=x-xminxmax-xmin

where *x* is the original feature value in the embedding vector, and $x_{\max}$ and $x_{\min}$ denote the maximum and minimum values of that feature type in the training set.

### 2.3 The DeepProSite framework

**Figure 1.**
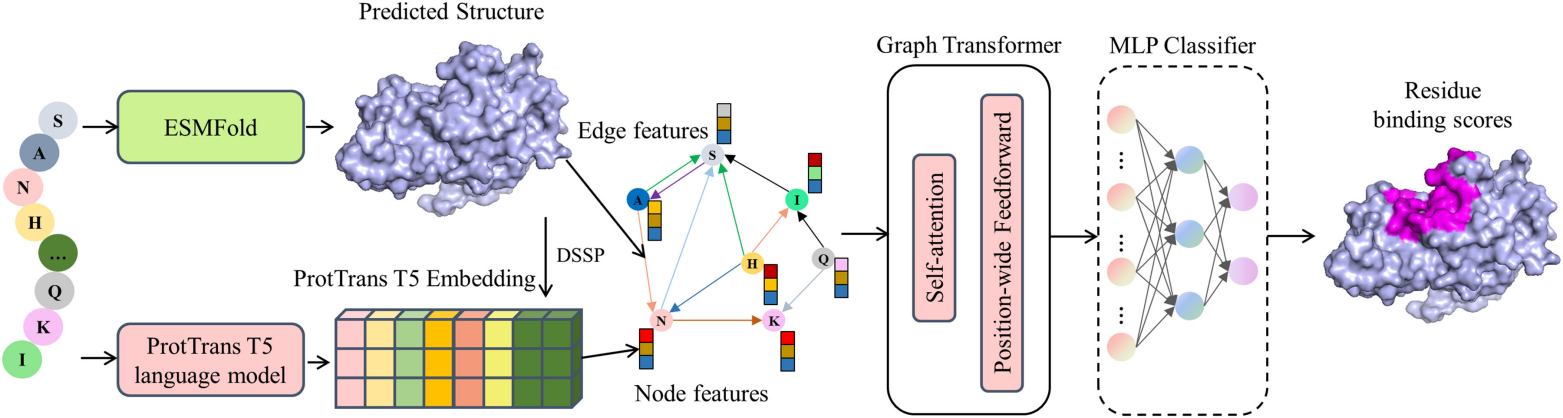
The overall pipeline of the presented DeepProSite method. The protein sequences are fed into ESMFold and ProtT5 pretrained language model to obtain both predicted structures and sequence embeddings. From the predicted structures, we build *k*-nearest neighbour graphs where each node’s position is determined by the α-carbon atom coordinate. The final node features consist of concatenated language model embeddings and DSSP, while several edge features account for the distance, direction, and orientation between adjacent nodes. A Graph Transformer model is then applied to aggregate the features of neighbouring nodes and edges and update the target node’s representation. This process ultimately captures the binding patterns of the proteins and proteins/peptides.

This work presents a new sequence-based method, DeepProSite, to improve protein binding site prediction by integrating protein spatial information. As illustrated in Fig. 1, the protein sequences are input to the ESMFold and ProtT5 pretrained language models to generate predicted protein structures and sequence embeddings, respectively. From the predicted structures, we constructed *k*-nearest neighbour graphs in which each node’s location is determined by the coordinate of the α-carbon atom, and *k *=* *30 in all experiments. The language model sequence embeddings and DSSP are concatenated as the final node features, and several edge features are computed end to end to reflect the distance, direction, and orientation between two adjacent nodes. The Graph Transformer model is applied to pay attention to and aggregate the features of neighbouring nodes and edges to update the target node’s representation, finally capturing the protein binding patterns.

#### 2.3.1 Geometric edge features

In addition to the precomputed node features (i.e. ProtT5 and DSSP) in the protein graph, we also adopted the relative spatial and positional encodings proposed in Ingraham *et al.* (2019) to represent the geometric edge features of two neighbouring residues, which were extracted end to end in our framework. We first augmented the coordinate of a residue $x_{i}$ with a local coordinate system $O_{i}$ to reflect the protein backbone geometry:


(2) Oi=bi ni bi×ni

where $b_{i}$ is the negative bisector of the angle between vectors $\left(x_{i-1}-x_{i}\right)$ and $\left(x_{i+1}-x_{i}\right)$, and $n_{i}$ is a unit vector normal to this plane. Concretely, we defined:


(3) vi=xi-xi-1xi-xi-1, bi=vi-vi+1vi-vi+1,  ni=vi×vi+1vi×vi+1

Finally, the rotationally and translationally invariant spatial edge features $e_{ij}^{\left(s\right)}$ are defined as follows:


(4) eijs=rxj-xi, OiTxj-xixj-xi, qOiTOj

where the first feature is a radial-based distance encoding (we apply 16 anisotropic Gaussian RBFs spaced from 0 to 20 Å), the second feature represents the relative direction of the neighbour node $x_{j}$ to the coordinate system of the centre node $x_{i}$, and the third feature is a quaternion representation of the spatial rotation matrix $O_{i}^{T}O_{j}$ to reflect orientation (Huynh 2009). In addition, to reflect the distances between residues in the sequence, we also concatenated the relative positional encodings $e_{ij}^{\left(p\right)}$ to the edge features using a sinusoidal function of the sequence gap between nodes *i* and *j*.

#### 2.3.2 Graph Transformer

The standard Transformer encoder includes two components: multi-head self-attention and position-wise feed-forward network. The self-attention module globally calculates the attention scores for all nodes to the target node and then aggregates them to update the target node. To incorporate protein structure information, GraphSite (Yuan *et al.* 2022) utilizes a *k*-nearest mask based on the distance matrix to exclude amino acids that are spatially remote when calculating attention scores. However, this framework cannot capture the geometric edge features between residues. Here, we integrated the edge features when updating the target node by


(5) hi′=hi+∑j∈Ni∪iαijWVhj∥eij

where the attention coefficients $\alpha_{ij}$ are computed via


(6) αij=softmaxWQhiTWKhj∥eijd

where $h_{i}$, $h_{j}$, and $h_{i}^{\prime }$ are the hidden embeddings of node *i*, node *j*, and the updated node *i*, respectively. $W_{Q}$, $W_{K}$, and $W_{V}$ are learnable weight matrices used to project the embeddings to the query, key, and value embeddings. *d* represents the hidden dimension, and ∥ represents the vector concatenation operation.

#### 2.3.3 Multilayer perceptron

The multilayer perceptron (MLP) leverages the output from the last layer of the Graph Transformer to estimate the residuewise probability of protein–protein/peptide binding via


(7) Y'=SigmoidHLW+b

where $H^{\left(L\right)}\in R^{n\times d}$ is the output hidden state of the *L*^th^ Graph Transformer layer; $W\in R^{d\times 1}$ is the learnable weight matrix; $b\in R^{d}$ is the bias term, and $Y^{'}\in R^{n\times 1}$ denotes the predictive scores of *n* residues in a query protein. The sigmoid function converts the raw network outputs into protein/peptide-binding probabilities between 0 and 1.

### 2.4 Implementation details

To evaluate our model’s performance, we randomly divided our training samples into five parts and employed the 5-fold cross-validation (CV) process. In each round, we trained a model on four parts of the data while using the remaining part for model evaluation. We repeated this procedure five times and used the mean validation performance to choose the model’s hyperparameters (Supplementary Tables S1–S4). To make predictions for the test set, we used all five trained models in the above CV steps for making inferences, and the average of these predictions was taken as the final prediction value of DeepProSite.

Finally, we utilized a four-layer graph transformer model with four attention heads, 64 hidden units, and a dropout rate of 0.2. The Adam optimizer with *β*_1_ of 0.9, *β*_2_ of 0.98, and *ε* of $10^{-9}$ was used to optimize our model using binary cross-entropy loss. In each training epoch, we fit our model in batches with 32 samples on 5000 proteins drawn from the training data using random sampling with replacement. DeepProSite was trained for 30 epochs with early stopping of eight epochs. To enhance the generalization and robustness of the model, we add random Gaussian noise to the features.

### 2.5 Evaluation metrics

The distribution of positive and negative samples in the dataset used here is highly unbalanced. To comprehensively evaluate the effectiveness of the proposed method, we used a range of standard evaluation metrics, including accuracy (ACC), precision (Pre), recall rate (Rec), specificity (Spe), *F*1 score (*F*1), Matthews correlation coefficient (MCC), area under the receiver operating characteristic curve (AUC), and area under the precision-recall curve (AUPRC). The formulas for computing these metrics are as follows:


(8) $$\mathrm{ACC}=\;\frac{\mathrm{TP}+\mathrm{TN}}{\mathrm{TP}+\mathrm{TN}+\mathrm{FP}+\mathrm{FN}}$$

(9) $$\mathrm{Precision}=\;\frac{\mathrm{TP}}{\mathrm{TP}+\mathrm{FP}}$$

(10) $$\mathrm{Recall}=\;\frac{\mathrm{TP}}{\mathrm{TP}+\mathrm{FN}}$$

(11) $$\mathrm{Specificity}=\;\frac{\mathrm{TN}}{\mathrm{TN}+\mathrm{FP}}$$

(12) $$F1=\;2\times \frac{\mathrm{Precision}\times \mathrm{Recall}}{\mathrm{Precision}+\mathrm{Recall}}$$

(13) $$\mathrm{MCC}=\;\frac{\mathrm{TP}\times \mathrm{TN}-\mathrm{FN}\times \mathrm{FP}}{\sqrt{\left(\mathrm{TP}+\mathrm{FP})\times (\mathrm{TP}+\mathrm{FN})\times (\mathrm{TN}+\mathrm{FP})\times (\mathrm{TN}+\mathrm{FN}\right)}}$$

where true positives (TP), true negatives (TN), false positives (FP), and false negatives (FN) represent the number of correctly predicted binding sites, correctly predicted nonbinding sites, incorrectly predicted binding sites, and incorrectly predicted nonbinding sites, respectively. AUC and AUPRC are threshold-independent metrics that provide a comprehensive evaluation of model performance. In contrast, other metrics require the use of a threshold and the conversion of predicted probabilities to binary predictions. The optimal threshold is determined by maximizing the MCC for each model. AUPRC is used for hyperparameter selection because it is more sensitive than other selectors, provides a comprehensive analysis when dealing with imbalanced datasets, and emphasizes few classes in imbalanced binary classification problems.

## 3 Results

### 3.1 Geometry feature improves the model performance

**Figure 2.**
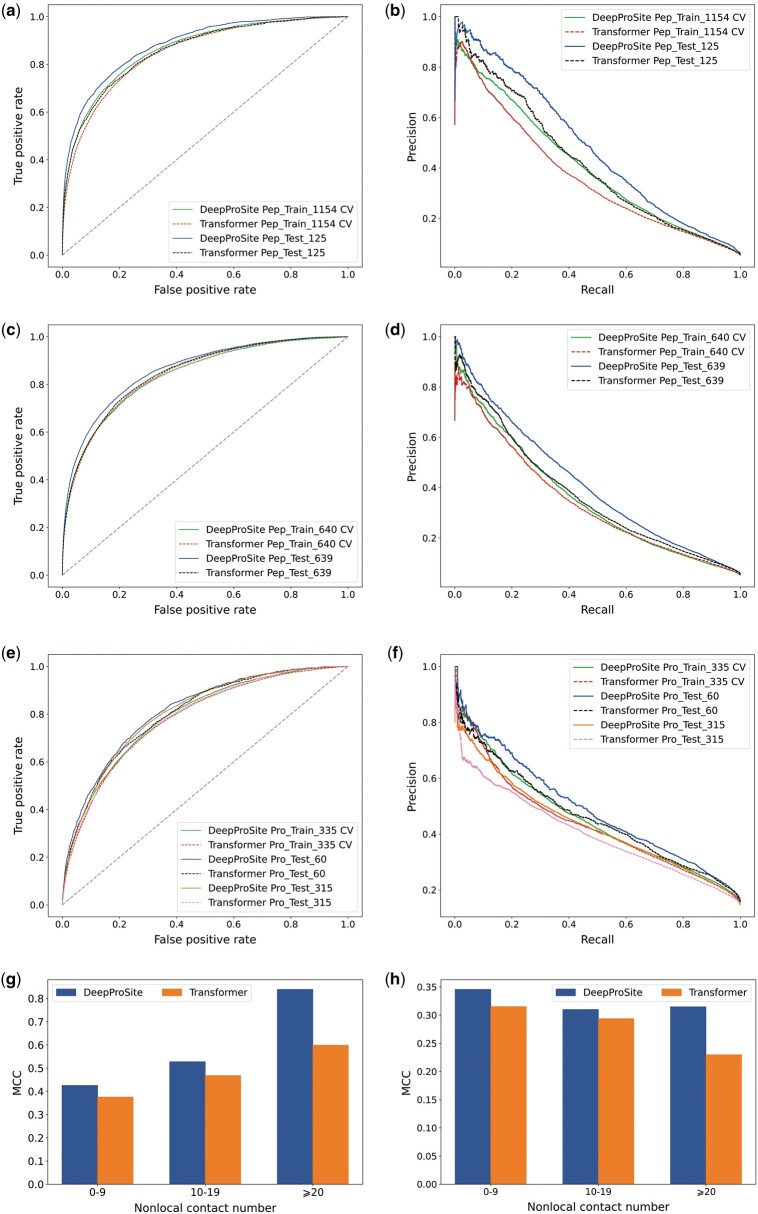
Impact of geometry and sequential information on model performance on datasets. Receiver operating characteristic curves and precision-recall curves of DeepProSite and Transformer on (a and b) Pep_Train_1154, Pep_Test_125, (c and d) Pep_Train_640, Pep_Test_639, and (e and f) Pro_Train_335, Pro_Test_60, Pro_Test_315. MCC comparison of two methods on amino acids with different nonlocal contacts in (g) Pep_Test_125 and (h) Pro_Test_315.

To evaluate the effectiveness of DeepProSite, we conducted AUC and AUPRC assessments employing both 5-fold CV and independent testing approaches. Specifically, on Pep_Train_1154 and Pep_Test_125, DeepProSite obtained AUCs of 0.864 and 0.883, respectively, and AUPRCs of 0.404 and 0.480, respectively (Supplementary Table S1). On Pep_Train_640 and Pep_Test_639, DeepProSite obtained AUCs of 0.842 and 0.861 and AUPRCs of 0.360 and 0.411, respectively (Supplementary Table S2). On Pro_Train_335 and Pro_Test_60, DeepProSite obtained AUCs of 0.795 and 0.813 and AUPRCs of 0.458 and 0.490, respectively (Supplementary Table S3). The consistency of our model’s performance on the CV and in independent testing proved its robustness. We compared DeepProSite to Transformer, a baseline model agnostic to geometry features (using the same hyperparameters as DeepProSite), to further investigate the benefits of adding protein geometry information and Graph Transformer. The input features of both models are identical, and the baseline model serves as a geometry-agnostic point of reference, enabling assessment of how spatial information affects the prediction of binding residues. According to our results, DeepProSite consistently outperforms the Transformer baseline model, as seen by greater *F*1, MCC, AUC, and AUPRC values on Pep_Test_125, Pep_Test_639, Pro_Test_60, and Pro_Test_315 (Supplementary Table S5). Precision-recall and ROC curves of DeepProSite and Transformer for both the 5-fold CV and independent datasets are shown in Fig. 2a–f, where the DeepProSite curve consistently lies above the Transformer curve. According to our findings, our method performs better than Transformer, possibly due to its ability to capture spatial information more effectively. The Graph Transformer operates by analyzing the relationships between nodes in graph data. This approach enables Graph Transformer to better handle structured data, such as molecular or protein structures.

To gain further insight into our approach and Transformer, we tested them on amino acids with various numbers of nonlocal contacts. Two residues with a sequence separation of more than 20 residues, yet the distance of their Cα atoms was less than 12 Å, are considered to be nonlocal contacts. On independent test datasets, as shown in Fig. 2g and h, our method consistently outperforms Transformer. Furthermore, the performance gap between the two methods widens significantly as the number of nonlocal contacts increases. Specifically, DeepProSite outperforms Transformer by 13.3% in terms of MCC for amino acids with 0–9 nonlocal contacts, as observed in Pep_Test_125. However, this gap widened to 40.1% for amino acids with ≥20 nonlocal contacts (Fig. 2g). Similarly, in Pro_Test_315, DeepProSite outperforms Transformer by 9.7% in MCC on amino acids with 0–9 nonlocal contacts, and the gap widens to 37.1% for amino acids with ≥20 nonlocal contacts (Fig. 2h). We observed similar trends in Pep_Test_639 and Pro_Test_60 (Supplementary Fig. S1). These findings emphasize the significance of spatial information and demonstrate DeepProSite’s efficiency in identifying protein/peptide-binding residues, by exploiting knowledge of protein structure.

### 3.2 Feature importance and model ablation

To evaluate the relative importance of the features utilized in our study, we conducted an investigation on the effect of different feature combinations. This process involved analyzing the performance of DeepProSite when each feature is used individually or in combination with other features. Specifically, we evaluated DeepProSite on Pep_Train_1154 and Pep_Test_125 using seven feature combinations, namely, (i) DSSP, (ii) ProtT5, (iii) DSSP + ProtT5, (iv) EVO (PSSM + HMM), (v) DSSP + EVO, (vi) ProtT5 + EVO, and (vii) DSSP + ProtT5 + EVO. As shown in Table 2, only the sequence representation extracted from ProT5 yielded satisfactory performance, with average AUCs of 0.858 and 0.880 and average AUPRCs of 0.386 and 0.467 on Pep_Train_1154 (CV) and Pep_Test_125, respectively. Alternatively, when relying only on DSSP as node features, the performance of our method declines, with average AUC decreases of 0.086 and 0.073 and average AUPRC decreases of 0.131 and 0.143, respectively. This decline indicates that while the structural properties of amino acids can provide some insight into the peptide binding sites, they are not sufficient to capture the complicated patterns involved. When integrating ProtT5 and DSSP, the average AUCs obtained on Pep_Train_1154 and Pep_Test_125 were 0.864 and 0.883, respectively, and the average AUPRCs obtained were 0.404 and 0.480, respectively, bringing a substantial improvement, indicating that the combined feature groups are not redundant. To determine whether evolutionary information contributes to the performance of our model, we compared the performance of replacing ProtT5 with evolutionary information, as well as adding additional evolutionary information. The method for extracting evolutionary information is described in Supplementary Section S1. The results show that replacing ProtT5 with evolutionary information becomes less effective, indicating the superiority of the language model in this method. In addition, the integration of all three features does not lead to a significant improvement, indicating that the embedding of the ProtT5 language model is likely to have captured evolutionary information. Considering the highest accuracy of ProtT5+DSSP, we chose ProtT5+DSSP as the final model. We also evaluated the performance of our model by using predicted protein structures from AlphaFold2. As shown in Supplementary Table S6, our method achieved similar results when using protein structures predicted from AlphaFold2 and ESMFold. We used ESMFold to predict protein structure because it offers rapid computational speed. Furthermore, we investigated different strategies for selecting adjacent residues based on the defined contact distance and the fixed *k*-nearest neighbours used in this study. The results show that the performance of the two strategies is similar (Supplementary Table S7). In addition, we investigate the impact of the attention mechanism [Equations (5) and (6)]. According to the results in Supplementary Table S8, the model’s performance with attention is better than that without attention.

**Table 2.** Comparison of feature performance in predicting PBPs on Pep_Train_1154 and Pep_Test_125.^a^

Feature	Pep_Train_1154 CV		Pep_Test_125	
	AUC	AUPRC	AUC	AUPRC
EVO	0.831	0.323	0.855	0.426
DSSP	0.778	0.273	0.810	0.337
ProtT5	0.858	0.386	0.880	0.467
ProtT5+EVO+DSSP	0.861	0.402	0.875	0.470
EVO+DSSP	0.835	0.342	0.852	0.427
ProtT5+EVO	0.862	0.386	**0.883**	0.477
ProtT5+DSSP (DeepProSite)	**0.864**	**0.404**	**0.883**	**0.480**

a EVO means evolutionary features represented by HMM and PSSM.

Bold fonts indicate the best results.

### 3.3 Comparison with state-of-the-art methods on peptide datasets

To assess the predictive capability of DeepProSite, we conducted a comparative analysis considering various other sequence-based and structure-based methods. Specifically, we evaluated the performance of DeepProSite on Pep_Test_125 and Pep_Test_639, and compared it against five sequence-based methods (SPRINT-Seq, PepBind, Visual, PepNN-Seq, and PepBCL) and four structure-based methods [PepSite (Petsalaki *et al.* 2009), Peptimap, SPRINT-Str (Taherzadeh *et al.* 2018), and PepNN-Struct (Abdin *et al.* 2020)]. Notably, for most of the compared methods, the source code is not available, and therefore, we obtained the results directly from the referenced studies. We also emphasize that predicting peptide-binding sites is an unbalanced prediction task where negative samples are more prevalent in the dataset. Thus, we placed more focus on evaluation metrics that consider both negative and positive samples, such as F1, MCC, AUC, and AUPRC. The results demonstrate that DeepProSite outperforms all other methods significantly (Tables 3 and 4). Specifically, on Pep_Test_125, DeepProSite achieves an MCC of 0.451 and an AUC of 0.883, exhibiting a relative improvement of 17.1% and 8.3%, respectively, compared to the second-best sequence-based method, PepBCL. Similar observations were made on Pep_Test_639, where DeepProSite outperforms PepBCL by 27.2% and 7.1% in terms of MCC and AUC, respectively. In addition, our method outperforms most other methods with a recall of 0.392 and precision of 0.578 on Pep_Test_125, and a recall of 0.400 and precision of 0.460 on Pep_Test_639. Recall and precision are imbalanced measures that heavily depend on the chosen threshold. Despite being a sequence-based predictor that solely relies on protein sequences, DeepProSite outperforms PepNN-Struct, a state-of-the-art structure-based method, in terms of both MCC and AUC by 40.5% (31.9%) and 5.0% (2.7%), respectively, on two independent test datasets (Tables 3 and 4). These results indicate that our method can accurately identify peptide-binding sites using only protein sequence information.

**Table 3.** Performance comparison of DeepProSite with state-of-the-art methods on Pep_Test_125 dataset.^a^

Method	Spe	Rec	Pre	MCC	AUC
Pepsite	0.970	0.180		0.200	0.610
Peptimap	0.950	0.320		0.270	0.630
SPRINT-Seq	0.960	0.210		0.200	0.680
SPRINT-Str	0.980	0.240		0.290	0.780
Visual	0.680	0.670		0.170	0.730
PepBind		0.344	0.469	0.372	0.793
PepNN-Seq				0.278	0.805
PepNN-Struct				0.321	0.841
PepBCL	**0.984**	0.315	0.540	0.385	0.815
DeepProSite	0.983	**0.392**	**0.578**	**0.451**	**0.883**

a Predictions of other methods are obtained from the corresponding publications.

**Table 4.** Performance comparison of DeepProSite with state-of-the-art methods on Pep_Test_639 dataset.

Method	Spe	Rec	Pre	MCC	AUC
PepBind		0.317	0.450	0.348	0.767
PepNN-Seq				0.251	0.792
PepNN-Struct				0.301	0.838
PepBCL	**0.983**	0.252	**0.470**	0.312	0.804
DeepProSite	0.972	**0.400**	0.460	**0.397**	**0.861**

### 3.4 Comparison with state-of-the-art methods on protein datasets

**Figure 3.**
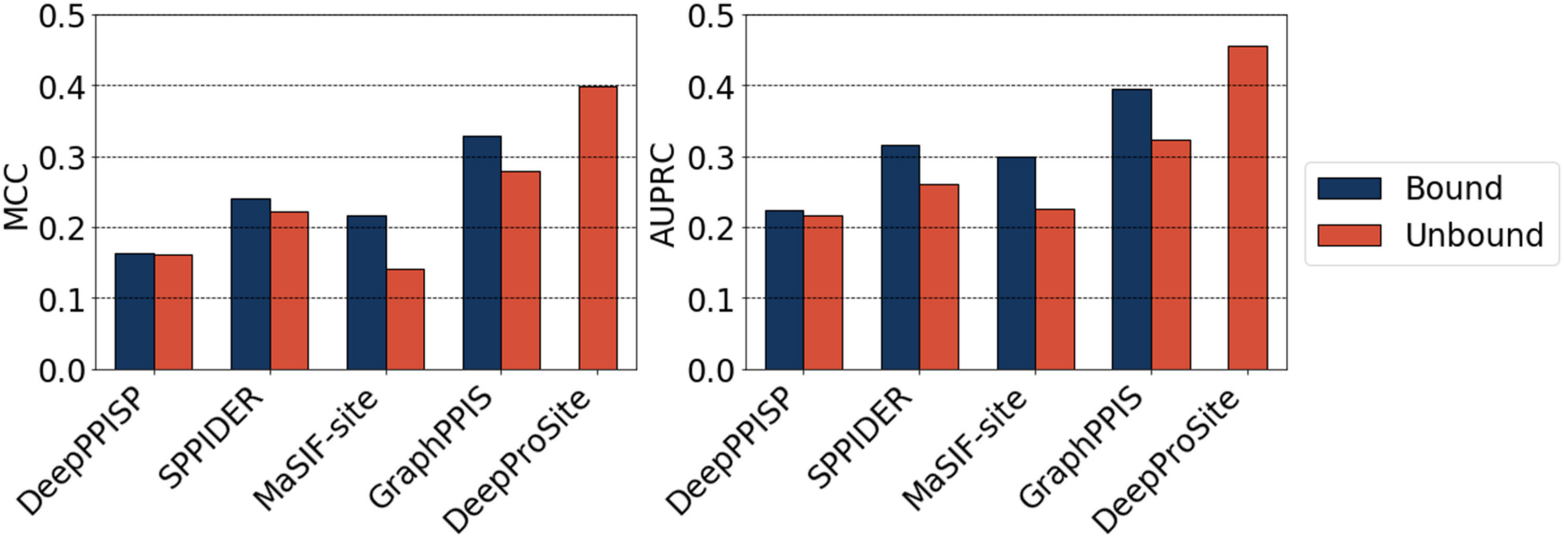
Performance comparison of DeepProSite with structure-based methods on 31 proteins with bound and unbound structures.

We compared the performance of DeepProSite with that of state-of-the-art methods on two protein datasets: Pro_Test_60 and Pro_Test_315. The methods evaluated included five sequence-based methods [PSIVER (Murakami and Mizuguchi 2010), SCRIBER (Zhang and Kurgan 2019), DLPred (Zhang *et al.* 2019), ProNA2020 (Qiu *et al.* 2020), and DELPHI (Li *et al.* 2021)] and five structure-based methods [SPPIDER (Porollo and Meller 2007), DeepPPISP (Zeng *et al.* 2020), MaSIF-site (Gainza *et al.* 2020), GraphPPIS (Yuan *et al.* 2021), and RGN (Wang *et al.* 2022)]. As shown in Table 5, DeepProSite exhibited superior performance compared to all other methods evaluated, even those that relied on the utilization of native protein structures in their structure-based approaches. Specifically, on Pro_Test_60, DeepProSite consistently outperformed PSIVER, ProNA2020, SCRIBER and DLPred on MCC and AUC, and outperformed DELPHI by 68.4% and 16.3%, respectively. DeepProSite also outperforms the aforementioned state-of-the-art structure-based methods and outperforms RGN by 8.6% and 2.8% on MCC and AUC, respectively. Pro_Test_315 is a recently solved protein dataset, on which DeepProSite achieved an MCC of 0.355 and an AUPRC of 0.432, surpassing DeepPPISP, SPPIDER, and MaSIF-site. Furthermore, DeepProSite outperforms the structure-based method GraphPPIS by 7.9% and 2.1% in terms of MCC and AUPRC, respectively (Table 6). Given that the training set for our method was originally constructed using native complex structures, it is valuable to explore the impact of utilizing unbound structures on predictive performance. Toward this goal, we conducted a comparison of DeepProSite’s performance in predicting a subset of Pro_Test_60 (bound) and its corresponding unbound structure against other structure-based methods. Because all four structure-based algorithms were trained with bound structures, their performance was found to be poor in predicting unbound structures, as shown in Fig. 3. In particular, the MaSIF-site’s MCC exhibited a 35.0% decrease and GraphPPIS’s MCC exhibited a 14.6% decrease, whereas DeepProSite was not affected by the induced fitting due to its unbiased training process using only sequence data (Supplementary Table S9). This outcome demonstrates DeepProSite as a more robust predictor than the other methods. Although we did not use evolutionary information or native structures, we still achieved competitive results with the latest structure-based methods ScanNet (Tubiana *et al.* 2022) and PeSTo (Krapp *et al.* 2023) (Supplementary Table S10). Taken together, these results demonstrate that our proposed method is practical and efficient, especially in situations where only the protein sequence is available rather than the native structure.

**Table 5.** Performance comparison of DeepProSite with state-of-the-art methods on Pro_Test_60 dataset.

Method	ACC	Rec	Pre	F1	MCC	AUC	AUPRC
PSIVER	0.561	0.534	0.188	0.278	0.074	0.573	0.190
ProNA2020	0.738	0.402	0.275	0.326	0.176	N/A	N/A
SCRIBER	0.667	0.568	0.253	0.350	0.193	0.665	0.278
DLPred	0.682	0.565	0.264	0.360	0.208	0.677	0.294
DELPHI	0.697	0.568	0.276	0.372	0.225	0.699	0.319
DeepPPISP	0.657	0.539	0.243	0.335	0.167	0.653	0.276
SPPIDER	0.752	0.557	0.331	0.415	0.285	0.755	0.373
MaSIF-site	0.780	0.561	0.370	0.446	0.326	0.775	0.439
GraphPPIS	0.776	0.584	0.368	0.451	0.333	0.786	0.429
RGN	0.785	**0.587**	0.382	0.463	0.349	0.791	0.441
DeepProSite	**0.842**	0.443	**0.501**	**0.470**	**0.379**	**0.813**	**0.490**

Note that all the other methods’ results are directly obtained from the previous works, GraphPPIS and RGN, as all these methods use the same training and testing datasets.

**Table 6.** Performance comparison of DeepProSite with state-of-the-art methods on Pro_Test_315 dataset.

Method	ACC	Rec	Pre	F1	MCC	AUC	AUPRC
DeepPPISP	0.603	0.622	0.206	0.310	0.157	0.660	0.256
SPPIDER	0.744	0.613	0.305	0.407	0.294	0.783	0.376
MaSIF-site	0.764	0.589	0.322	0.417	0.304	0.778	0.372
GraphPPIS	0.739	**0.689**	0.313	0.430	0.329	0.798	0.423
DeepProSite	**0.804**	0.576	**0.378**	**0.457**	**0.355**	**0.805**	**0.432**

### 3.5 Influence of predicted protein structure quality

**Figure 4.**
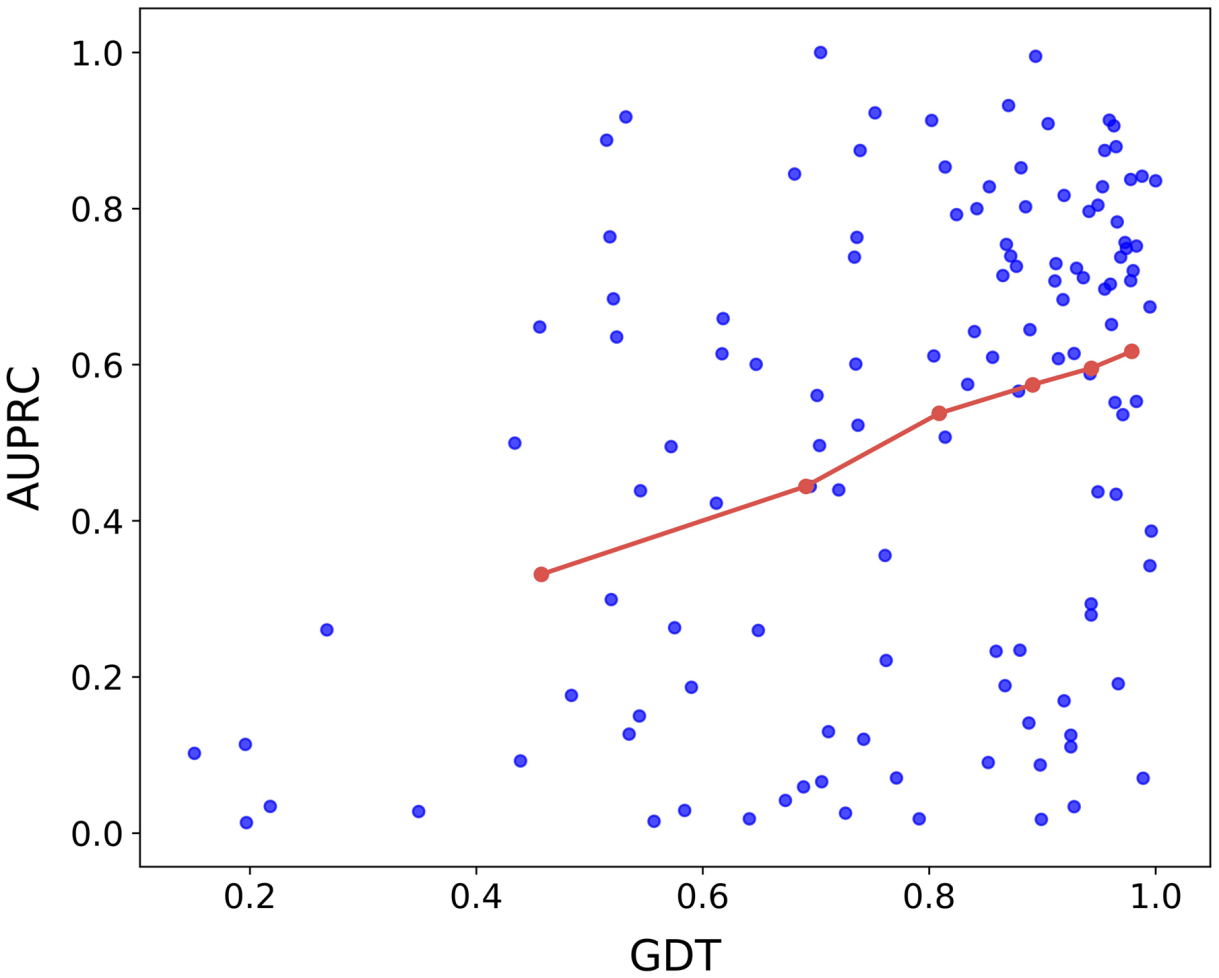
Positive correlation between the predicted quality of ESMFold as measured by GDT and the performance of DeepProSite as measured by AUPRC on Pep_Test_125. Each protein’s corresponding GDT and AUPRC values are denoted by blue scatter points, and a red line represents the average GDT and AUPRC for each bin after sorting all proteins according to GDT and dividing them into six bins.

DeepProSite relies on predicted protein structures for geometric deep learning, making the accuracy of ESMFold prediction crucial for downstream binding site prediction. The average global distance test (GDT) (Zemla 2003) between the predicted structures generated by ESMFold and the corresponding native structures in Pep_Test_125 was calculated using SPalign (Yang *et al.* 2012) to assess the overall quality of protein structures. Figure 4 displays the quality of protein structures on Pep_Test_125, as well as the corresponding AUPRC values for each protein (blue scatterplots). Specifically, we divided proteins in Pep_Test_125 into six bins based on their GDT scores and computed the average GDT and AUPRC for each bin (red line). The results showed a positive correlation between the prediction quality of ESMFold as assessed by GDT and the performance of DeepProSite, as measured by AUPRC. According to predictions made by DeepProSite, the top 30% of proteins with the highest GDT (average GDT = 0.964) have an average AUPRC of 0.627, which is significantly higher than that of the bottom 30% of proteins with the lowest GDT (average GDT = 0.526), which showed an average AUPRC of 0.375 according to Mann–Whitney *U*-test results (*P-*value = 1.40 × 10^−4^) . Moreover, our analysis revealed a negative correlation between the prediction error of ESMFold at the amino acid level, as measured by the distance between predicted and natural amino acids after structure alignment, and the performance of DeepProSite (Supplementary Tables S11–S14). Overall, these results highlight the critical role of accurate protein structure prediction in successfully predicting protein/peptide binding sites in DeepProSite.

### 3.6 Case study

**Figure 5.**
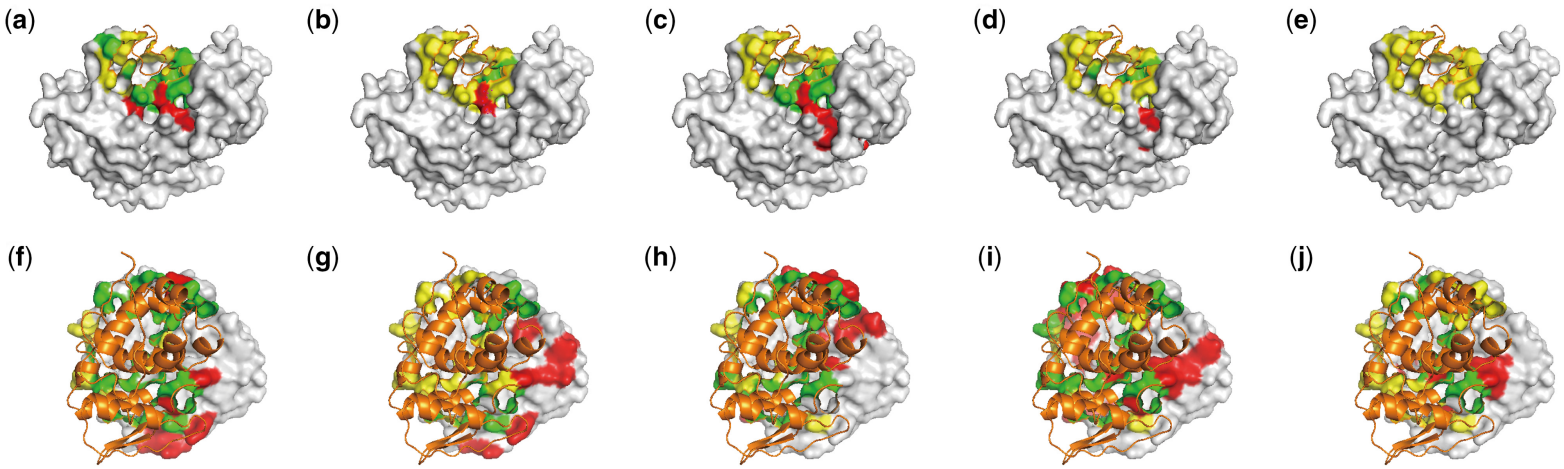
Visualization of predicted binding residues for two cases predicted by DeepProSite and other methods. The results predicted by DeepProSite (a), the geometric diagnostic baseline method Transformer (b), PepNN-Struct (c), PepBCL (d), and PepBind (e) are shown for the first protein (PDB ID: 4L3O, chain A) from Pep_Test_125. The results predicted by DeepProSite (f), Transformer (g), GraphPPIS (h), SPPIDER (i) and ProNA2020 (j) are shown for the second protein (PDB ID: 4BVX, chain A) from Pro_Test_315. The TP, FP, and FN are colored in green, red, and yellow, respectively.

To visually depict DeepProSite’s predicted ability, two protein sequences, PDB ID: 4L3O (chain A) and 4BVX (chain A), were randomly selected from the Pep_Test_125 and Pro_Test_315 datasets for illustrative purposes. Figure 5 shows the results of predicting binding sites with different methods, including DeepProSite, Transformer (a geometry-independent baseline method), and two distinct methods for predicting peptide binding sites, namely PepBCL and PepBind, along with two methods, SPPIDER and ProNA2020, used for predicting protein binding sites. In the case of protein 4L3O_A, among the total of 297 residues, 28 residues are peptide-binding. DeepProSite correctly predicted 16 true positive binding residues out of the 19 predicted binding residues, resulting in F1 score, MCC, and AUPRC of 0.638, 0.622, and 0.707, respectively. In contrast, Transformer predicted only five correct binding residues, resulting in a lower F1 score, MCC, and AUPRC of 0.326, 0.294, and 0.576, respectively, without the use of a graph. PepNN-Struct predicted 11 correct binding residues, resulting in *F*1 score, MCC, and AUPRC of 0.458, 0.419, and 0.492, respectively, even as a structure-based prediction method. Similar results were obtained for PepBCL and PepBind. In protein 4BVX_A, there are a total of 203 residues with 33 protein binding residues. DeepProSite predicted 34 binding residues, 25 of which are true positives, resulting in an *F*1 score of 0.758, MCC of 0.711, and AUPRC of 0.754, whereas Transformer predicted only 10 correct binding residues, resulting in a lower *F*1 score, MCC, and AUPRC of 0.507, 0.410, and 0.470, respectively. GraphPPIS predicted 24 correct binding residues, resulting in *F*1 score, MCC and AUPRC of 0.676, 0.610, and 0.612, respectively, even as a structural prediction method. Similar results were obtained for SPPIDER and ProNA2020. The results indicate that incorporating the spatial information captured from the graph module can enhance the precision of our approach in recognizing binding sites and minimizing the chances of false positives. As shown in Fig. 5a, the majority of the false positive binding residues that are identified by DeepProSite (highlighted in red) are located at or near the protein–protein/peptide interaction interface or near the protein/peptide structure.

### 3.7 Extending DeepProSite to other types of ligands

To investigate the generalizability of our model, we analyzed how DeepProSite performed when detecting various binding sites for different ligands. We retrained DeepProSite on datasets of different ligand types and compared it with the results of various methods, such as TargetS (Yu *et al.* 2013), S-SITE (Yang *et al.* 2013b), COACH (Yang *et al.* 2013b), IonCom (Hu *et al.* 2016), ATPbind (Hu *et al.* 2018), DELIA (Xia *et al.* 2020), and GraphBind (Xia *et al.* 2021), on multiple benchmark ligand datasets, including five biologically relevant molecules, namely DNA, RNA, ATP, HEME, and carbohydrate, as well as three metal ions, specifically Mg^2+^, Ca^2+^, and Mn^2+^. Details of these benchmark datasets are provided in Supplementary Section S4 and Table S15. The performance of DeepProSite compared to multiple methods is shown in Supplementary Tables S16 and S17. In each dataset, DeepProSite outperforms competing methods in most metrics. Table 7 displays the results for Rec, Pre, F1, MCC, and AUC metrics achieved by DeepProSite and GraphBind. The results show that DeepProSite improves the MCC and AUC by 0.023–0.107 and 0.011–0.068 for DNA, RNA, ATP, Mg^2+^, Ca^2+^, and Mn^2+^, respectively, compared with GraphBind. The above results indicate that DeepProSite has strong robustness and generalization ability, and can serve as a reliable tool for predicting ligand binding sites based on protein sequences.

**Table 7.** Performance comparison of DeepProSite and GraphBind on seven ligand-binding test sets.^a^

	Rec		Pre		F1		MCC		AUC	
	*G*	*D*	*G*	*D*	*G*	*D*	*G*	*D*	*G*	*D*
DNA_Test_129	**0.676 ± 0.027**	0.634 ± 0.016	0.425 ± 0.017	**0.513 ± 0.014**	0.522 ± 0.005	**0.567 ± 0.003**	0.499 ± 0.004	**0.540 ± 0.003**	0.927 ± 0.006	**0.939 ± 0.001**
RNA_Test_117	0.463 ± 0.036	**0.493 ± 0.044**	0.294 ± 0.017	**0.312 ± 0.023**	0.358 ± 0.008	**0.380 ± 0.008**	0.322 ± 0.008	**0.347 ± 0.004**	0.854 ± 0.006	**0.860 ± 0.002**
ATP_Test_41	0.603 ± 0.037	**0.678 ± 0.030**	0.666 ± 0.035	**0.700 ± 0.039**	0.631 ± 0.012	**0.687 ± 0.007**	0.616 ± 0.011	**0.674 ± 0.008**	0.939 ± 0.006	**0.956 ± 0.002**
HEM_Test_96	**0.775 ± 0.032**	0.676 ± 0.025	0.610 ± 0.026	**0.656 ± 0.024**	**0.682 ± 0.008**	0.665 ± 0.006	**0.661 ± 0.008**	0.640 ± 0.007	**0.962 ± 0.003**	0.958 ± 0.002
Mg^2+^_Test_651	**0.259 ± 0.013**	0.251 ± 0.019	0.410 ± 0.026	**0.482 ± 0.038**	0.317 ± 0.006	**0.328 ± 0.009**	0.320 ± 0.007	**0.342 ± 0.004**	0.827 ± 0.007	**0.854 ± 0.002**
Ca^2+^_Test_515	0.325 ± 0.031	**0.369 ± 0.014**	0.563 ± 0.040	**0.606 ± 0.024**	0.410 ± 0.017	**0.458 ± 0.005**	0.420 ± 0.011	**0.466 ± 0.004**	0.863 ± 0.012	**0.883 ± 0.001**
Mn^2+^_Test_144	0.563 ± 0.044	**0.582 ± 0.024**	0.626 ± 0.030	**0.649 ± 0.031**	0.591 ± 0.012	**0.613 ± 0.007**	0.588 ± 0.011	**0.610 ± 0.008**	0.951 ± 0.006	**0.953 ± 0.002**

a Predictions of competing methods are provided through GraphBind. Bold values indicate performance that is better than the compared tool. G, GraphBind; D, DeepProSite.

### 3.8 Establishment of a web server to facilitate the prediction of multiple types of binding sites

**Figure 6.**
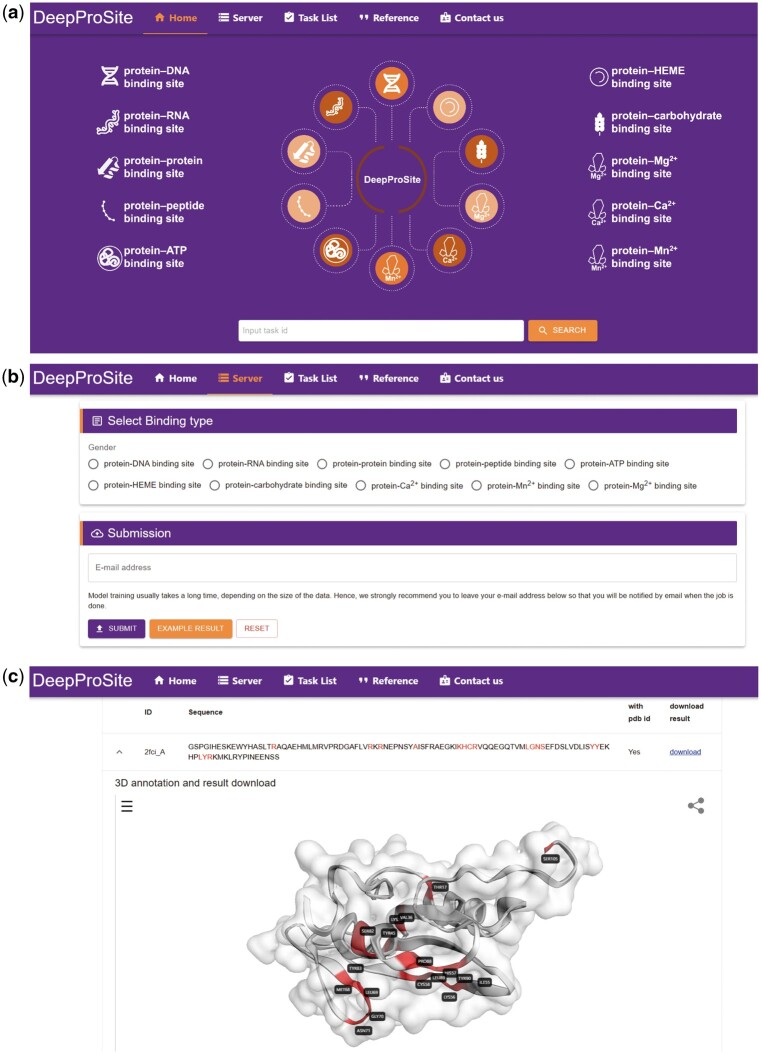
User interface of the DeepProSite web service. (a and b) Users can enter protein sequences in FASTA format and select the preferred model. (c) The prediction results can be visualized for proteins available in PDB. Prediction results can also be downloaded as text file.

To facilitate the use of the proposed DeepProSite, we developed a public web server dedicated to predicting various binding sites in protein sequences. The web server can be accessed via https://inner.wei-group.net/DeepProSite/. Figure 6 displays an instruction manual for the web server. Specifically, users can readily submit one or more protein sequences in FASTA format and choose from a range of available prediction models, including protein–DNA binding site prediction, protein–RNA binding site prediction, protein–protein binding site prediction, protein–peptide binding site prediction, and other ligand-specific binding site prediction, such as ATP, HEME, and metal ion prediction. The predictions can be visualized for proteins available in PDB. The predictions can also be produced as downloadable text.

## 4 Conclusion

Deep learning methods for predicting protein binding sites provide advantages that are not available in biological experiments for understanding biological activity and drug design. Unfortunately, current prediction methods that depend on sequence analysis only consider adjacent contextual features in sequences, resulting in inadequate predictive performance. Additionally, methods based on protein structure are often not applicable to a wide range of proteins due to inadequate information on their tertiary structures.

Here, we present a topology-aware Graph Transformer-based model, DeepProSite, which utilizes ESMFold-predicted tertiary structures and pretrained protein language models for protein binding site prediction. By relying solely on protein sequences, DeepProSite achieves excellent predictive performance, surpassing even the most advanced structure-based methods, thus overcoming the limitations associated with both existing sequence-based and structure-based methods. We also show that when predicting unbound structures, the accuracy of competing structure-based methods largely decreases, whereas DeepProSite maintains comparable performance as a predictor based solely on sequence, further demonstrating the benefit of the unbiased training process using sequence only. In summary, the superior performance of DeepProSite compared to the state of the art is attributed to three factors: (i) the high-quality structure predicted by ESMFold, (ii) the pretrained protein language model offers a powerful representation that enhances the quality of predictions, and (iii) the structure-aware Graph Transformer effectively identifies and predicts binding residue patterns, thus contributing to the overall efficiency of the approach.

Despite the benefits of the DeepProSite method, certain aspects of our approach can be improved. For example, by utilizing protein primary sequences to construct heterogeneity maps, the model’s robustness to varying structure prediction qualities can be enhanced. In addition, our method is limited to identifying possible protein binding residues based only on protein-related information, and cannot predict the binding pattern of specific ligands. These issues will be improved in future work.

In summary, our method can provide valuable insights for studying protein–protein/peptide binding patterns, pathogenic mechanisms of mutations, and drug development. For instance, some diseases are caused by mutations resulting in alterations in protein–protein/peptide binding sites, which lead to abnormal protein functions. Understanding the characteristics of these sites and their effects upon mutation can help reveal these pathogenic mechanisms. In addition, the prediction of protein–protein/peptide binding sites can provide valuable information for drug development, including designing more precise targets and improving drug selectivity and affinity. Such predictions can also be used to study protein interaction networks and biological signalling to further understand the biological functions of proteins. In the future, we intend to enhance the Graph Transformer’s design and incorporate multitask learning to expand its application to various other domains. This involves predicting the binding sites of proteins with other ligands and identifying functional sites of proteins, such as methylation sites, phosphorylation sites, and allosteric sites.

## Supplementary Material

btad718_Supplementary_Data

## Data Availability

The DeepProSite web server can be accessed at https://inner.wei-group.net/DeepProSite/. The datasets and source codes of DeepProSite are available at https://github.com/WeiLab-Biology/DeepProSite.
