# Supplementary material for: DeepProSite: structure-aware protein binding site prediction using ESMFold and pretrained language model
Source: Bioinformatics. 2023 Nov 28;39(12):btad718. doi: 10.1093/bioinformatics/btad718 (PMC10723037; doi:10.1093/bioinformatics/btad718)
Supplement: btad718_Supplementary_Data [file btad718_supplementary_data.pdf]

Supplementary Information

DeepProSite: structure-aware protein binding site prediction using ESMFold and pretrained language model

Yitian Fang[1,2], Yi Jiang[3], Leyi Wei[4], Qin Ma[3], Zhixiang Ren[2], Qianmu Yuan[5,*] & Dong-Qing Wei[1,2,*]

[1] State Key Laboratory of Microbial Metabolism, Shanghai-Islamabad-Belgrade Joint Innovation Center on Antibacterial Resistances, Joint International Research Laboratory of Metabolic & Developmental Sciences and School of Life Sciences and Biotechnology, Shanghai Jiao Tong University, Shanghai 200040, China

[2] Peng Cheng Laboratory, Shenzhen 518055, China

[3] Department of Biomedical Informatics, College of Medicine, The Ohio State University, Columbus, OH, 43210, USA

[4] School of Software, Shandong University, Jinan 250100, China

[5] School of Computer Science and Engineering, Sun Yat-sen University, Guangzhou 510000, China

* To whom correspondence should be addressed.

E-mail: dqwei@sjtu.edu.cn or yuanqm3@mail2.sysu.edu.cn

Supplementary section 1. Evolutionary information

Evolutionarily conserved residues may have critical effects on important protein properties, including protein structure, function, subcellular localization, and interaction (Chothia *et al.* 2003; Gough *et al.* 2001; Jin *et al.* 2009). Among them, conserved residues usually form key domains in proteins, such as helix-turn-helix motif, zinc finger, leucine zipper, and beta-alpha-beta motif (Aravind *et al.* 2005; Chothia and Lesk 1986; Klug 2010; Laity *et al.* 2001; Landschulz *et al.* 1988; Laskowski *et al.* 1996; Matthews and Sunde 2002; Pauling *et al.* 1951; Vinson *et al.* 1989). To investigate whether ProtT5 has captured evolutionary traits, we also tested the widely used position-specific scoring matrix (PSSM) and hidden Markov model (HMM) profile. Specifically, PSSM was generated by running PSI-BLAST (Altschul *et al.* 1997) against the UniRef 90 database (Suzek *et al.* 2007) with three iterations and an E-value of 0.001. The HMM profile was created by HHblits with default parameters (Remmert *et al.* 2012) to align the query sequence against the UniClust30 database (Mirdita *et al.* 2017). Each residue is embedded into a 20-dimensional vector through PSSM and HMM respectively, representing the frequencies of 20 amino acids occurring at the corresponding position. Values in PSSM and HMM are normalized to scores between 0 and 1 using Formula (1), where x is the original feature value in the embedding vector, and x_{max} and x_{min} denote the maximum and minimum values of that feature type in the training set.

$$x_{norm} = \frac{x - x_{min}}{x_{max} - x_{min}} \#(1)$$

Supplementary section 2. Details of positional encoding

We implemented position encoding for edges with the relative sequence position encoding, which is the same as in Transformer (Vaswani *et al.* 2017), as shown below:

$$PE_{(pos,2i)} = \sin(pos/10000^{2i/d_{model}}) \#(2)$$

$$PE_{(pos,2i+1)} = cos(pos/10000^{2i/d_{model}}) \#(3)$$

where *pos* is the edge relative sequence position, *i* is the dimension, and d_{model} is the dimension of the embedding.

Supplementary section 3. Details of training procedure

About optimizing schedule

We use the Adam optimizer, where the learning rate changes as the number of training iterations increases during the training process. The concrete formula for updating learning rate is shown as the following:

$$lrate = d_{model}^{-0.5} \cdot min(step\_num^{-0.5}, step\_num \cdot warmup\_steps^{-1.5}) \#(4)$$

In the above formula, *lrate* is the learning rate, and the maximum learning rate is 0.0004. d_{model} refers to the dimension of the token embedding, *step_num* refers to the training iterations, and *warmup_steps* refers to a hyperparameter. According to the formula, we can know that the learning rate will increase when the *step_num* is less than *warmup_steps* and will decrease when

the *step_num* is over than *warmup_steps*.

About runtime

DeepProSite is implemented in Python v3.7.11. The neural network is built based on PyTorch v1.8.0 (Paszke *et al.* 2019). The model is trained with a Tesla V100 (32G) GPU. Training a model with a dataset of 923 proteins typically takes around 13GB of memory and 50 minutes to complete 30 epochs.

Supplementary section 4. Generalization test data of DeepProSite model

The DNA-binding protein datasets (DNA_Train_573 and DNA_test_129) and RNA-binding protein datasets (RNA_Train_495 and RNA_test_117) were obtained from GraphBind (Xia *et al.* 2021) and constructed from the BioLiP database (Yang *et al.* 2012). These datasets were divided into a training set and a test set according to the release date. A residue is considered a binding residue if the minimum atomic distance between the target residue and the nucleic acid molecule is less than 0.5 Å, in addition to the sum of the van der Waal's radius of the two nearest atoms. The datasets containing proteins that bind to Ca^{2+}, Mn^{2+}, Mg^{2+}, and HEME were obtained from DELIA (Xia *et al.* 2020). Protein chains released before January 6, 2016, were assigned to the training sets, while the remaining chains were assigned to the test sets. CD-HIT was used to reduce sequence similarity to 30% for each ligand between any pair of proteins in the combined training and test sets. ATP_Train_388 and ATP_Test_41 were downloaded from ATPbind (Hu *et al.* 2018). These protein sequences were obtained from the PDB database. CD-HIT software was also used to remove redundancy in the dataset to ensure that sequence similarity in the dataset remained below 40%. For the prediction of protein–carbohydrate binding site, we used the same dataset as in previous studies: SPRINT-CBH (Taherzadeh *et al.* 2016) and StackCBPred (Gattani *et al.* 2019). A residue is defined as a carbohydrate-binding site if any atom in the residue is within 3.5 Å (Malik and Ahmad 2007) of any carbohydrate atom. The details of the training and test datasets for different ligands can be found in Supplementary Table S10.

(a) (b)

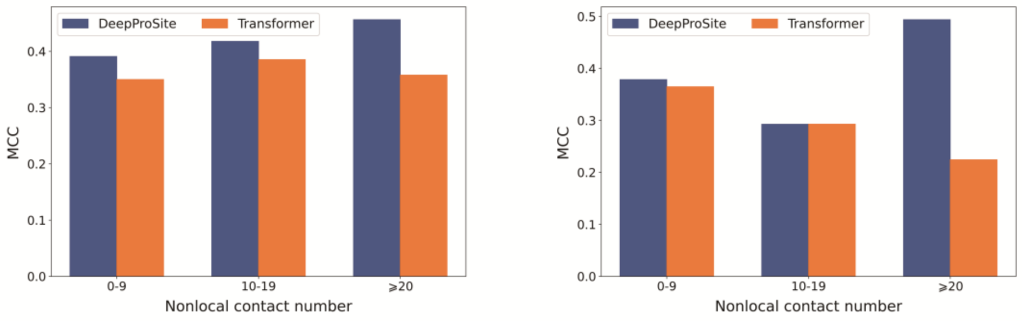

Figure S1. The MCC of DeepProSite and Transformer on amino acids that exhibit varying numbers of nonlocal contacts in (a) Pep_Test_639 and (b) Pro_Test_60.

Table S1. The performance of DeepProSite on the 5-fold cross-validation (CV) and test dataset of Pep_Train_1154 and Pep_Test_125 using different hyperparameters.

Hyperparameter	Value	5-Fold CV AUC	5-Fold CV AUPRC	Test AUC	Test AUPRC
edge_features	**16**	**0.864**	**0.404**	**0.883**	**0.480**
	32	0.860	0.397	0.878	0.464
	64	0.859	0.385	0.879	0.467
	128	0.859	0.388	0.878	0.466
hidden_dim	16	0.849	0.350	0.857	0.417
	32	0.861	0.380	0.867	0.445
	**64**	**0.864**	**0.404**	**0.883**	**0.480**
	128	0.854	0.401	0.882	0.471
num_encoder_layers	2	0.860	0.393	0.870	0.452
	3	0.863	0.397	0.874	0.457
	**4**	**0.864**	**0.404**	**0.883**	**0.480**
augment_eps	0	0.861	0.394	0.876	0.464
	0.01	0.861	0.398	0.878	0.464
	0.05	**0.865**	0.401	0.882	0.474
	**0.1**	0.864	**0.404**	**0.883**	**0.480**
dropout	0.1	0.855	0.387	0.878	0.468
	0.2	0.859	0.395	**0.883**	**0.481**
	**0.3**	**0.864**	**0.404**	**0.883**	0.480
batch_size	4	0.857	0.387	0.876	0.460
	16	0.861	0.401	**0.884**	**0.483**
	**32**	**0.864**	**0.404**	0.883	0.480
	64	0.862	0.395	0.877	0.467

Table S2. The performance of DeepProSite on the 5-fold CV and test dataset of Pep_Train_640 and Pep_Test_639 using different hyperparameters.

Hyperparameter	Value	5-Fold CV AUC	5-Fold CV AUPRC	Test AUC	Test AUPRC
edge_features	16	**0.843**	0.351	0.857	0.400
	**32**	0.842	**0.360**	**0.861**	**0.411**
	64	0.839	0.346	0.857	0.394
	128	0.837	0.344	0.854	0.389
hidden_dim	16	0.838	0.327	0.854	0.379
	32	**0.844**	0.345	0.860	0.400
	**64**	0.842	**0.360**	**0.861**	**0.411**
	128	0.837	0.335	0.855	0.396
num_encoder_layers	2	0.836	0.344	0.859	0.396
	**3**	**0.842**	**0.360**	**0.861**	**0.411**
	4	0.837	0.348	0.858	0.408
augment_eps	0	**0.843**	0.350	0.859	0.406
	0.01	0.840	0.355	**0.861**	**0.411**
	0.05	**0.843**	0.356	**0.861**	0.410
	**0.1**	0.842	**0.360**	**0.861**	**0.411**
Dropout	0.1	0.833	0.334	0.855	0.387
	0.2	0.833	0.339	0.857	0.401
	**0.3**	**0.842**	**0.360**	**0.861**	**0.411**
batch_size	4	0.837	0.340	0.855	0.389
	**16**	**0.842**	**0.360**	**0.861**	**0.411**
	32	0.840	0.349	0.856	0.410
	64	**0.842**	0.337	0.858	0.399

Table S3. The performance of DeepProSite on the 5-fold CV and test dataset of Pro_Train_335 and Pro_Test_60 using different hyperparameters.

Hyperparameter	Value	5-Fold CV AUC	5-Fold CV AUPRC	Test AUC	Test AUPRC
edge_features	**16**	**0.795**	**0.458**	**0.813**	**0.490**
	32	0.794	**0.458**	0.809	0.474
	64	**0.795**	0.455	0.810	0.479
	128	0.787	0.438	0.803	0.460
hidden_dim	16	0.785	0.416	0.797	0.452
	32	**0.803**	0.457	0.810	0.479
	**64**	0.795	**0.458**	**0.813**	**0.490**
	128	0.792	0.439	0.808	0.484
num_encoder_layers	2	**0.796**	0.448	0.812	0.477
	3	0.794	0.450	0.808	0.489
	**4**	0.795	**0.458**	**0.813**	**0.490**
augment_eps	0	0.791	0.446	0.807	0.476
	0.01	0.793	0.443	0.810	0.486
	0.05	0.792	0.445	0.807	0.482
	**0.1**	**0.795**	**0.458**	**0.813**	**0.490**
Dropout	0.1	0.791	0.442	**0.816**	**0.491**
	0.2	0.793	0.441	0.804	0.471
	**0.3**	**0.795**	**0.458**	0.813	0.490
batch_size	4	0.798	0.455	0.808	0.479
	16	**0.801**	0.454	0.809	0.488
	**32**	0.795	**0.458**	**0.813**	**0.490**
	64	0.796	0.454	0.810	0.478

Table S4. The performance of DeepProSite on the 5-fold cross-validation (CV) and test dataset of Pep_Train_1154 and Pep_Test_125 using different numbers of *k*-nearest neighbors.

The number of *k*-nearest neighbors	5-Fold CV AUC	5-Fold CV AUPRC	Test AUC	Test AUPRC
5	0.845	0.364	0.858	0.435
10	0.850	0.376	0.869	0.445
15	0.856	0.388	0.876	0.470
20	0.860	0.390	0.879	0.464
25	0.860	0.393	0.879	0.470
**30**	**0.864**	**0.404**	**0.883**	**0.480**
35	0.863	0.398	0.881	0.466
40	0.860	0.394	0.876	0.466

Table S5. Performance comparison with the geometric-agnostic baseline model Transformer on Pep_Test_125, Pep_Test_639, Pro_Test_60 and Pro_Test_315. The highest values are bolded.

Dataset	Method	Spe	Rec	Pre	F1	MCC	AUC	AUPRC	ACC
Pep_	Transformer	0.965	**0.441**	0.423	0.432	0.398	0.857	0.417	0.936
Test_125	DeepProSite	**0.983**	0.392	**0.578**	**0.467**	**0.451**	**0.883**	**0.480**	**0.950**
Pep_	Transformer	0.964	0.392	0.395	0.394	0.357	0.847	0.369	0.932
Test_639	DeepProSite	**0.972**	**0.400**	**0.460**	**0.428**	**0.397**	**0.861**	**0.411**	**0.940**
Pro_	Transformer	0.859	**0.550**	0.422	**0.478**	0.369	0.801	0.461	0.810
Test_60	DeepProSite	**0.917**	0.443	**0.501**	0.470	**0.379**	**0.813**	**0.490**	**0.842**
Pro_	Transformer	**0.854**	0.518	0.373	0.433	0.326	0.785	0.402	**0.806**
Test_315	DeepProSite	0.842	**0.576**	**0.378**	**0.457**	**0.355**	**0.805**	**0.432**	0.804

Note: ACC stands for binary acc.

Table S6. The predictive performance of DeepProSite on Pep_Test_125 when using different predicted structures.

Structural information	Spe	Rec	Pre	F1	MCC	AUC	AUPRC	ACC
AlphaFold2 predicted structures	**0.985**	0.383	**0.603**	**0.468**	**0.457**	**0.888**	**0.482**	**0.952**
ESMFold predicted structures	0.983	**0.392**	0.578	0.467	0.451	0.883	0.480	0.950

Table S7. Performance comparison of fixed distance and fixed number on Pep_Test_125.

Neighbors selection	Spe	Rec	Pre	F1	MCC	AUC	AUPRC	ACC
C_α atoms <10 Å	0.979	0.408	0.539	0.464	0.442	0.879	0.464	0.948
k-nearest neighbors	**0.983**	**0.392**	**0.578**	**0.467**	**0.451**	**0.883**	**0.480**	**0.950**

Table S8. The ablation study on attention mechanism on Pep_Test_125.

Structural information	Spe	Rec	Pre	F1	MCC	AUC	AUPRC	ACC
DeepProSite w/o attention	**0.985**	0.353	**0.586**	0.440	0.430	0.870	0.457	**0.950**
DeepProSite	0.983	**0.392**	0.578	**0.467**	**0.451**	**0.883**	**0.480**	**0.950**

Note: w/o represents without the corresponding module.

Table S9. Performance comparison of DeepProSite with other structure-based methods on 31 proteins with bound and unbound structures.

Method	Bound		Unbound	
	MCC	AUPRC	MCC	AUPRC
DeepPPISP	0.163	0.223	0.162	0.217
SPPIDER	0.240	0.315	0.222	0.260
MaSIF-site	0.217	0.299	0.141	0.225
GraphPPIS	0.328	0.395	0.280	0.323
DeepProSite			**0.398**	**0.455**

Note: Predictions of competing methods are provided through the work of GraphPPIS (Yuan *et al.* 2021).

Table S10. Performance comparison of DeepProSite with other structure-based geometric deep learning methods on Pro_Test_45 dataset.

Method	Spe	Rec	Pre	F1	MCC	AUC	AUPRC	ACC
ScanNet	0.907	0.386	0.469	0.423	0.317	0.780	0.450	0.816
PeSTo	**0.947**	0.359	**0.588**	0.446	**0.376**	**0.794**	**0.510**	**0.844**
DeepProSite	0.886	**0.455**	0.467	**0.461**	0.345	0.778	0.452	0.808

Note: For fair comparison, sequences with similarity greater than 0.25 compared to ScanNet and PeSTo training sets in Pro_Test_60 and Pro_Test_315 were removed, resulting in an independent test dataset of Pro_Test_45.

Table S11. Model performance for amino acids with varying degrees of predicted errors. In Pep_Test_125, all amino acids were sorted according to the distance between the native and predicted amino acid and evenly divided into five bins. The median distances and AUPRCs were calculated from these bins.

Bin Range (Å)	0.01-0.38	0.38-0.66	0.66-1.21	1.21-4.16	>4.16
Median Distance (Å)	0.26	0.51	0.88	1.85	17.23
AUPRC	0.616	0.568	0.506	0.413	0.204

Table S12. Model performance for amino acids with varying degrees of predicted errors. In Pep_Test_639, all amino acids were sorted according to the distance between the native and predicted amino acid and evenly divided into five bins. The median distances and AUPRCs were calculated from these bins.

Bin Range (Å)	0.01-0.36	0.36-0.61	0.61-1.08	1.08-3.11	>3.11
Median Distance (Å)	0.25	0.48	0.80	1.59	11.16
AUPRC	0.508	0.476	0.419	0.387	0.212

Table S13. Model performance for amino acids with varying degrees of predicted errors. In Pro_Test_60, all amino acids were sorted according to the distance between the native and predicted amino acid and evenly divided into five bins. The median distances and AUPRCs were calculated from these bins.

Bin Range (Å)	0.02-0.32	0.32-0.50	0.50-0.81	0.81-1.77	>1.77
Median Distance (Å)	0.22	0.40	0.63	1.11	4.64
AUPRC	0.603	0.582	0.537	0.408	0.383

Table S14. Model performance for amino acids with varying degrees of predicted errors. In Pro_Test_315, all amino acids were sorted according to the distance between the native and predicted amino acid and evenly divided into five bins. The median distances and AUPRCs were calculated from these bins.

Bin Range (Å)	0.01-0.35	0.35-0.57	0.57-0.96	0.96-2.59	>2.59
Median Distance (Å)	0.24	0.45	0.73	1.39	10.45
AUPRC	0.486	0.480	0.440	0.399	0.384

Table S15. Summary of the eight benchmark datasets.

Type	Dataset	$N_{protein}$[a]	N_{pos}[b]	N_{neg}[c]	PNratio[d]
DNA	DNA_Train_573	573	14,479	145,404	0.100
	DNA_Test_129	129	2,240	35,275	0.064
RNA	RNA_Train_495	495	14,609	122,290	0.119
	RNA_Test_117	117	2,031	35,314	0.058
Ca^{2+}	CA_Train_1022	1,022	4,830	255,917	0.019
	CA_Test_515	515	2,958	186,678	0.016
Mn^{2+}	MN_Train_440	440	1,931	150,229	0.013
	MN_Test_144	144	612	50,838	0.012
Mg^{2+}	MG_Train_1194	1,194	4,147	320,736	0.013
	MG_Test_651	651	2,321	244,088	0.010
HEME	HEM_Train_175	175	3,851	44,477	0.087

	HEM_Test_96	96	2,012	26,341	0.076
ATP	ATP_Train_388	388	5,657	142,086	0.040
	ATP_Test_41	41	674	14,159	0.048
carbohydrate	CBH_Train_100	100	1028	25958	0.040
	CBH_Test_49	49	508	13230	0.038

[a] Number of proteins.

[b] Number of binding residues.

[c] Number of nonbinding residues.

[d] PNratio = N_{pos}/N_{neg}.

Table S16. Performance comparison of DeepProSite with other state-of-the-art methods on test datasets of biologically relevant molecules (DNA, RNA, ATP and HEME) and metal ions (Mg^{2+}, Ca^{2+}, and Mn^{2+}).

Dataset	Method	Rec	Pre	F1	MCC	AUC
DNA_Test_129	TargetDNA	0.417	0.280	0.335	0.291	0.825
	TargetS	0.239	0.370	0.291	0.262	N/A
	DNAPred	0.396	0.353	0.373	0.332	0.845
	SVMnuc	0.316	0.371	0.341	0.304	0.812
	COACH-D	0.324	0.360	0.341	0.302	0.761
	NucBind	0.323	0.373	0.346	0.309	0.797
	DNABind	0.601	0.346	0.440	0.411	0.858
	GraphBind	**0.676**	0.425	0.522	0.499	0.927
		**±0.027**	±0.017	±0.005	±0.004	±0.006
	DeepProSite	0.634	**0.513**	**0.567**	**0.540**	**0.939**
		±0.016	**±0.014**	**±0.003**	**±0.003**	**±0.001**
RNA_Test_117	RNABindRPlus	0.273	0.227	0.248	0.202	0.717
	SVMnuc	0.231	0.240	0.235	0.192	0.729
	COACH-D	0.221	0.252	0.235	0.195	0.663
	NucBind	0.231	0.235	0.233	0.189	0.715
	aaRNA	0.484	0.166	0.247	0.214	0.771
	NucleicNet	0.371	0.201	0.261	0.216	0.788
	GraphBind	0.463	0.294	0.358	0.322	0.854
		±0.036	±0.017	±0.008	±0.008	±0.006
	DeepProSite	**0.493**	**0.312**	**0.380**	**0.347**	**0.860**
		**±0.044**	**±0.023**	**±0.008**	**±0.004**	**±0.002**
ATP	TargetS	0.516	0.689	0.590	0.580	N/A
(ATP_Test_41)	S-SITE	0.570	0.505	0.536	0.513	0.801
	COACH	0.632	0.703	0.666	0.652	N/A

	ATPbind	0.631	0.756	**0.688**	0.677	0.915
	DELIA	0.642	**0.758**	0.695	**0.685**	0.947
	GraphBind	0.603	0.666	0.631	0.616	0.939
		±0.037	±0.035	±0.012	±0.011	±0.006
	DeepProSite	**0.678**	0.700	0.687	0.674	**0.956**
		**±0.030**	±0.039	±0.007	±0.008	**±0.002**
HEME	TargetS	0.493	**0.756**	0.597	0.588	N/A
(HEM_Test_96)	S-SITE	0.619	0.580	0.599	0.568	0.813
	COACH	0.677	0.403	0.505	0.476	0.835
	DELIA	0.648	0.660	0.654	0.628	0.951
	GraphBind	**0.775**	0.610	**0.682**	**0.661**	**0.962**
		**±0.032**	±0.026	**±0.008**	**±0.008**	**±0.003**
	DeepProSite	0.676	0.656	0.665	0.640	0.958
		±0.025	±0.024	±0.006	±0.007	±0.002
Mg^{2+}	TargetS	0.154	0.449	0.229	0.259	N/A
(MG_Test_651)	S-SITE	0.243	0.132	0.171	0.169	0.682
	COACH	**0.273**	0.124	0.171	0.169	0.675
	IonCom	0.155	0.317	0.208	0.217	0.685
	DELIA	0.143	0.562	0.228	0.280	0.780
	GraphBind	0.259	0.410	0.317	0.320	0.827
		±0.013	±0.026	±0.006	±0.007	±0.007
	DeepProSite	0.251	**0.482**	**0.328**	**0.342**	**0.854**
		±0.019	**±0.038**	**±0.009**	**±0.004**	**±0.002**
Ca^{2+}	TargetS	0.174	0.506	0.259	0.291	N/A
(CA_Test_515)	S-SITE	0.303	0.124	0.176	0.174	0.661
	COACH	0.297	0.162	0.210	0.203	0.671
	IonCom	0.190	0.331	0.241	0.242	0.717
	DELIA	0.182	0.556	0.274	0.313	0.795
	GraphBind	0.325	0.563	0.410	0.420	0.863
		±0.031	±0.040	±0.017	±0.011	±0.012
	DeepProSite	**0.369**	**0.606**	**0.458**	**0.466**	**0.883**
		**±0.014**	**±0.024**	**±0.005**	**±0.004**	**±0.001**
Mn^{2+}	TargetS	0.395	0.499	0.441	0.438	N/A
(MN_Test_144)	S-SITE	0.369	0.526	0.434	0.435	0.817
	COACH	0.562	0.272	0.367	0.381	0.821
	IonCom	0.531	0.495	0.512	0.506	0.872
	DELIA	0.513	0.632	0.566	0.565	0.903
	GraphBind	0.563	0.626	0.591	0.588	0.951

	±0.044	±0.030	±0.012	±0.011	±0.006
DeepProSite	**0.582**	**0.649**	**0.613**	**0.610**	**0.953**
	**±0.024**	**±0.031**	**±0.007**	**±0.008**	**±0.002**

Note: Predictions of competing methods are provided through the work of GraphBind.

Table S17. Comparisions of DeepProSite with SPRINT-CBH and StackCBPred on independent test dataset for protein-carbohydrate binding sites prediction.

Method	Spe	Rec	Pre	F1	MCC	AUC	AUPRC	ACC
SPRINT-CBH	0.925	-	-	-	0.195	-	-	0.906
StackCBPred	0.795	-	-	-	0.159	-	-	0.786
DeepProSite	**0.982**	0.382	0.450	0.413	**0.394**	0.854	0.339	**0.960**

Note: Predictions of competing methods are provided through the work of StackCBPred.

References

Altschul, S.F., Madden, T.L., Schaffer, A.A. *et al.* Gapped BLAST and PSI-BLAST: a new generation of protein database search programs. *Nucleic Acids Res.* 1997;**25**:3389-3402.

Aravind, L., Anantharaman, V., Balaji, S. *et al.* The many faces of the helix-turn-helix domain: transcription regulation and beyond. *FEMS Microbiol. Rev.* 2005;**29**:231-262.

Chothia, C., Gough, J., Vogel, C. *et al.* Evolution of the protein repertoire. *Science* 2003;**300**:1701-1703.

Chothia, C. and Lesk, A.M. The relation between the divergence of sequence and structure in proteins. *The EMBO journal* 1986;**5**:823-826.

Gattani, S., Mishra, A. and Hoque, M.T. StackCBPred: A stacking based prediction of protein-carbohydrate binding sites from sequence. *Carbohydr. Res.* 2019;**486**:107857.

Gough, J., Karplus, K., Hughey, R. *et al.* Assignment of homology to genome sequences using a library of hidden Markov models that represent all proteins of known structure. *J. Mol. Biol.* 2001;**313**:903-919.

Hu, J., Li, Y., Zhang, Y. *et al.* ATPbind: accurate protein–ATP binding site prediction by combining sequence-profiling and structure-based comparisons. *J. Chem. Inf. Model.* 2018;**58**:501-510.

Jin, J., Xie, X., Chen, C. *et al.* Eukaryotic protein domains as functional units of cellular evolution. *Science signaling* 2009;**2**:ra76-ra76.

Klug, A. The discovery of zinc fingers and their applications in gene regulation and genome manipulation. *Annual review of biochemistry* 2010;**79**:213-231.

Laity, J.H., Lee, B.M. and Wright, P.E. Zinc finger proteins: new insights into structural and functional diversity. *Curr. Opin. Struct. Biol.* 2001;**11**:39-46.

Landschulz, W.H., Johnson, P.F. and McKnight, S.L. The leucine zipper: a hypothetical structure common to a new class of DNA binding proteins. *Science* 1988;**240**:1759-1764.

Laskowski, R.A., Rullmann, J.A.C., MacArthur, M.W. *et al.* AQUA and PROCHECK-NMR: programs for checking the quality of protein structures solved by NMR. *J. Biomol. NMR* 1996;**8**:477-486.

Malik, A. and Ahmad, S. Sequence and structural features of carbohydrate binding in proteins and assessment of predictability using a neural network. *BMC Struct. Biol.* 2007;**7**:1-14.

Matthews, J.M. and Sunde, M. Zinc fingers--folds for many occasions. *IUBMB life* 2002;**54**:351-355.

Mirdita, M., Von Den Driesch, L., Galiez, C. *et al.* Uniclust databases of clustered and deeply annotated protein sequences and alignments. *Nucleic Acids Res.* 2017;**45**:D170-D176.

Paszke, A., Gross, S., Massa, F. *et al.* Pytorch: An imperative style, high-performance deep learning library. *Adv. Neural Inf. Process. Syst.* 2019;**32**.

Pauling, L., Corey, R.B. and Branson, H.R. The structure of proteins: two hydrogen-bonded helical configurations of the polypeptide chain. *Proceedings of the National Academy of Sciences* 1951;**37**:205-211.

Remmert, M., Biegert, A., Hauser, A. *et al.* HHblits: lightning-fast iterative protein sequence searching by HMM-HMM alignment. *Nat. Methods* 2012;**9**:173-175.

Suzek, B.E., Huang, H., McGarvey, P. *et al.* UniRef: comprehensive and non-redundant UniProt reference clusters. *Bioinformatics* 2007;**23**:1282-1288.

Taherzadeh, G., Zhou, Y., Liew, A.W.-C. *et al.* Sequence-based prediction of protein–carbohydrate binding sites using support vector machines. *J. Chem. Inf. Model.* 2016;**56**:2115-2122.

Vaswani, A., Shazeer, N., Parmar, N. *et al.* Attention is all you need. *Adv. Neural Inf. Process. Syst.* 2017;**30**.

Vinson, C.R., Sigler, P.B. and McKnight, S.L. Scissors-grip model for DNA recognition by a family of leucine zipper proteins. *Science* 1989;**246**:911-916.

Xia, C.-Q., Pan, X. and Shen, H.-B. Protein–ligand binding residue prediction enhancement through hybrid deep heterogeneous learning of sequence and structure data. *Bioinformatics* 2020;**36**:3018-3027.

Xia, Y., Xia, C.-Q., Pan, X. *et al.* GraphBind: protein structural context embedded rules learned by hierarchical graph neural networks for recognizing nucleic-acid-binding residues. *Nucleic Acids Res.* 2021;**49**:e51-e51.

Yang, J., Roy, A. and Zhang, Y. BioLiP: a semi-manually curated database for biologically relevant ligand–protein interactions. *Nucleic Acids Res.* 2012;**41**:D1096-D1103.

Yuan, Q., Chen, J., Zhao, H. *et al.* Structure-aware protein-protein interaction site prediction using deep graph convolutional network. *Bioinformatics* 2021;**38**:125-132.
